# Methods for reproductive tract scoring as a tool for improving sow productivity^1,2^

**DOI:** 10.1093/tas/txz160

**Published:** 2019-10-13

**Authors:** Matthew R Romoser, Benjamin J Hale, Jacob T Seibert, Tom Gall, Christopher J Rademacher, Kenneth J Stalder, Lance H Baumgard, Aileen F Keating, Jason W Ross

**Affiliations:** 1 Department of Animal Sciences, Iowa State University, Ames, IA; 2 TriOak Foods Inc., Oakville, IA; 3 College of Veterinary Medicine, Iowa State University, Ames, IA

**Keywords:** gilt, litter size, sow lifetime productivity, vulva width

## Abstract

Improving sow lifetime productivity is essential for maximizing farm profitability. Study objectives were to determine the accuracy for different vulva scoring methods in a commercial production system and to assess whether gilt reproductive tract scoring [evaluated by vulva width (VW)] prior to puberty could serve as useful gilt selection criteria. To accomplish this objective, 958 prepubertal replacement gilts in a commercial system were evaluated at approximately 15 wk of age. Gilt body weight (BW) was recorded in addition to 4 different methods to evaluate VW. Methods for VW assessment included digital caliper measurement (mm), visual evaluation and scoring by trained farm personnel [Farm Score (FS)], and 2 methods using scoring tools [Vulva Score Method A and B (VSA and VSB, respectively)] specifically calibrated from the VW distribution measured on gilts from previous studies. The VSA and FS methods assigned gilts to one of 3 categories (S, M, L, and 1, 2, 3, respectively) whereas VSB classified gilts vulvas using a 5-point scoring system (1 to 5). At 15 wk of age, a low proportion of variability in vulva size (27.8 ± 0.1 mm) could be explained by BW (62.2 ± 0.2 kg; *R*^2^ = 0.05). All 3 scoring methods were effective in categorizing gilts based upon VW, as the measured VW size within methods differed by score (*P* < 0.01). The proportion of gilts achieving their first parity increased with score for VSA (64.7%, 73.2%, and 84.4%; *P* = 0.02), VSB (66.0%, 71.7%, 79.2%, 76.4%, and 84.2%; *P* = 0.02), and FS (67.2%, 75.0%, and 88.8%; *P* = 0.03), but VSA, VSB, and FS did not influence percentage of gilts achieving their second parity (*P* = 0.32, 0.29, and 0.30, respectively). Litter performance of gilts scored as M or L using VSA improved with an increased total born over 2 parities compared to those scored as S (23.96 vs. 26.38 pigs; *P* < 0.01) as well as born alive (21.13 vs. 23.05 pigs; *P* < 0.05). Results were similar for VSB, where scores 2 to 5 had greater total born (23.97 vs. 26.33 pigs; *P* < 0.01) and born alive (21.11 vs. 23.02 pigs; *P* < 0.05) through 2 parities compared to gilts scored 1. Using the FS method, total born pigs tended to be increased (*P* = 0.06) through 2 parities for gilts having a 2 or 3 vulva score compared to those scored as a 1. Collectively, assessing VW at approximately 15 wk of age may identify sows with improved productivity through 2 parities as breeding herd females.

## INTRODUCTION

Accurate selection and retention of replacement females with the reproductive ability to wean a maximum number of quality pigs over recurrent parities is imperative for enhancing sow lifetime productivity (SLP; Stalder et al., 2003). However, selecting for SLP is arduous as reproductive performance is under the control of numerous genetic loci and largely impacted by environmental factors (Serenius and Stalder, 2006). Currently, the most predictive trait for identifying young females with the greatest potential for SLP is age at puberty. Puberty, or age at first estrus, is predictive of a sow’s ability to produce at least 3 parities (Patterson et al., 2010). Furthermore, gilts reaching puberty earlier are more likely to display visible estrus and ovulate within 10 d after weaning (Sterning et al., 1998), which reduces non-productive sow days in the breeding herd. However, identification of a gilt’s age of puberty in commercial systems is labor intensive and inefficient with most gilt development housing facilities.

Gilt management practices prior to their introduction into the breeding herd can ultimately impact female reproductive potential. During development, a gilt’s reproductive tract becomes responsive to hormonal changes and is associated with increased follicular development and total tract size at approximately 70 d of age (Dyck and Swierstra, 1983). This change in reproductive tract size is presumably the result of endogenous estrogen production from the initial follicular growth during the prepubertal period. Our previous research findings demonstrated that the initiation of tertiary follicle development is highly variable within a cohort of gilts but begins after postnatal day (PND) 75 (Graves et al., 2019). Additionally, reproductive tract growth [(assessed by vulva width (VW)] at PND 95 to 115 has predictive value because of its association with a gilt’s ability to achieve puberty by 200 d of age. Collectively, this led to our hypothesis that vulva scoring in a commercial pork production system, as assessed by VW at approximately 15 wk of age could effectively identify gilts with different reproductive potential. Study objectives were to evaluate different prepubertal vulva scoring methods on a commercial farm to determine the method’s effectiveness for identifying females having superior reproductive performance potential.

## MATERIALS AND METHODS

This study was conducted in cooperation with TriOak Foods, Inc. with animal procedures approved by the Iowa State University Animal Care and Use Committee.

### Animals

A total of 958 gilts [Landrace × Yorkshire (Topigs Norsvin, Vught, the Netherlands and Fast Genetics, Saskatchewan, Canada)] born across 3 consecutive birth weeks in late 2015 and weaned at 20 ± 3 d were used for this study. At approximately 6 wk of age, gilts were transferred from the parent multiplier to the initial receiving gilt development unit (GDU). At approximately 15 wk of age, gilts were subjected to routine culling/selection criteria (lameness, poor structural conformation, abdominal hernias). Following initial culling, the remaining gilts were transported to 2 separate GDUs specific to the destination breed-to-wean sow farm. The number shipped from each birth week depended on replacement gilt demand at the respective sow farm. As a result, the number of potential replacement gilts having the opportunity to enter the sow herd was reduced to 731.

### Gilt Development

At the initial receiving GDU, gilts were housed (providing approximately 0.84-m^2^ floor space per gilt) in groups of 25 on fully slatted floors. Upon arrival, gilts were acclimated by inoculation with porcine epidemic diarrhea virus, as well as vaccinating for ileitis (EnterisolIleitis, Boehringer Ingelheim) and erysipelas (Ery Vac, ARKO Laboratories). Once transported to the sow farm-specific GDU, all gilts were inoculated with a site-specific porcine reproductive and respiratory syndrome virus strain. At the sow farm, gilts were again allocated approximately 0.84-m^2^ floor space per head. At approximately 20 wk of age, gilts were again subjected to selection criteria unrelated to reproductive performance. At approximately 20 to 26 wk of age, gilts entered the designated sow farm with boar exposure beginning immediately using mature boars (>18 mo of age) which were rotated daily and not used more than once per week. Boar exposure (approximately 10 min of contact per pen) for puberty stimulation and heat detection was done via fence-line contact. Gilts not demonstrating behavioral estrus by 36 wk of age were culled, and their culling reason noted as failure to display estrus.

### Data Collection

**Figure 1.**
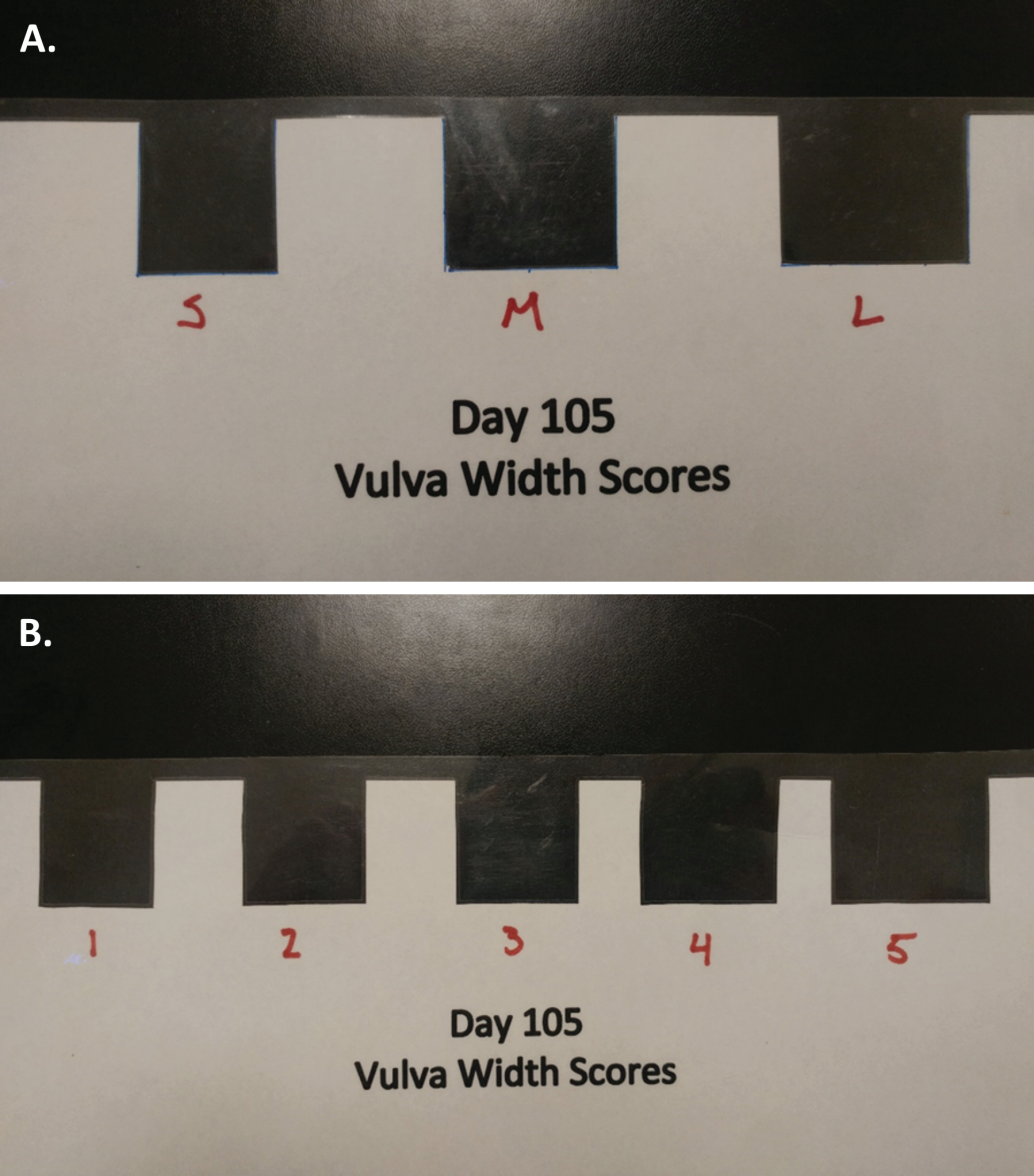
Vulva Score Method A and B (VSA and VSB) Tools. (A) VSA tool used to score gilts based on vulva width (VW): small (S; VW < 27 mm), medium (M; VW 27–34 mm), and large (L; VW > 34 mm). (B) VSB tool used to score gilts based on vulva width: 1 (VW < 27 mm), 2 (VW 27–29 mm), 3 (29–31 mm), 4 (VW 31–33 mm), and 5 (VW > 33 mm).

Data collection took place at the receiving GDU in March 2016. At approximately 15 wk of age, gilts were individually identified using an ear tag (Hog Max, Destron Fearing) and body weight (BW) was recorded. Vulva width (mm) was recorded in millimeters using Ultra Tech digital calipers (General Tools, Secaucus, NJ) with the guides of the digital calipers positioned at the widest part of the vulva. Vulva scores were assigned for each gilt using 3 different scoring methods. Vulva Score Method A (VSA) used a 3-score strategy designed using previous data (Graves et al., 2019) where gilts were stratified into the following categories; Small (S; VW < 27 mm), Medium (M; VW 27 to 34 mm), and Large (L; VW > 34 mm). Vulva Score Method B (VSB) used a similar tool to categorize gilts into 5 groups based on VW: 1 (VW < 27 mm), 2 (VW 27 to 29 mm), 3 (VW 29 to 31 mm), 4 (VW 31 to 33 mm), and 5 (VW > 33 mm). The design of the tool used for VSA and VSB was a laminated card with precisely sized series of sections removed to accurately assign a vulva score based on the above described dimensions (Fig. 1). Farm Score (FS) was a visual, subjective assessment of vulva size (conducted visually by trained farm staff) which stratified gilts into 3 categories (1, 2, or 3), where score 1 intended to identify gilts whose vulva size represented the bottom 15%, a score 2 was considered intermediate and represented the middle 70%, and a score 3 was intended to represent the 15% of gilts with the largest vulvas. The FS was conducted independently of all other vulva score measurements to avoid bias in the subjective score. At the sow farm, production data were recorded by farm staff, documented, and stored in an online swine database (PigKnows LLC.) and later merged with the prepubertal vulva size scores corresponding with each sow.

### Reproductive Performance

Production records of selected gilts were monitored and data were recorded for any event occurring prior to achieving first parity (P1), which included return to estrus events, failed pregnancy checks, abortion, and other reasons for removal. For those that successfully farrowed, litter data recorded included total piglets born (TB), number born alive (BA), stillborn (SB), and mummified fetuses (MM). Following P1 weaning, subsequent reproductive performance data were collected through the second parity (P2).

### Statistical Analysis

Statistical Analysis Systems University Edition, version 9.4 (Cary, NC) was used for all statistical analysis. Regression analyses (PROC REG, SAS v.9.4, SAS Inst. Inc., Cary, NC) were completed to evaluate the relationships between BW and VW measures and to generate coefficient of determination values. Group means for each fixed effect level were compared using PROC TTEST. A chi-square (χ ^2^) analysis was performed (PROC FREQ, SAS v.9.4) to estimate the association between vulva score classification and ability to achieve P1 and P2. Additionally, for each vulva scoring method (VSA, VSB, or FS) mixed model methods (PROC MIXED, SAS v.9.4) were used to analyze the litter performance data, with a model where the fixed effects were: vulva score, sow farm, birth week, and the associated interactions. The random error term was the only random effect included in any model used for analyses. Prior to analyzing litter performance data, data points extending beyond 2.5 SDs from the mean for TB, BA, SB, and MM were considered outliers and were removed from analysis. The number of outliers from any of the analyses ranged from 0 to 6 animals.

## RESULTS

### Vulva Scoring Methods Were Effective in Separating Groups of Gilts Based on Vulva Width

**Figure 2.**
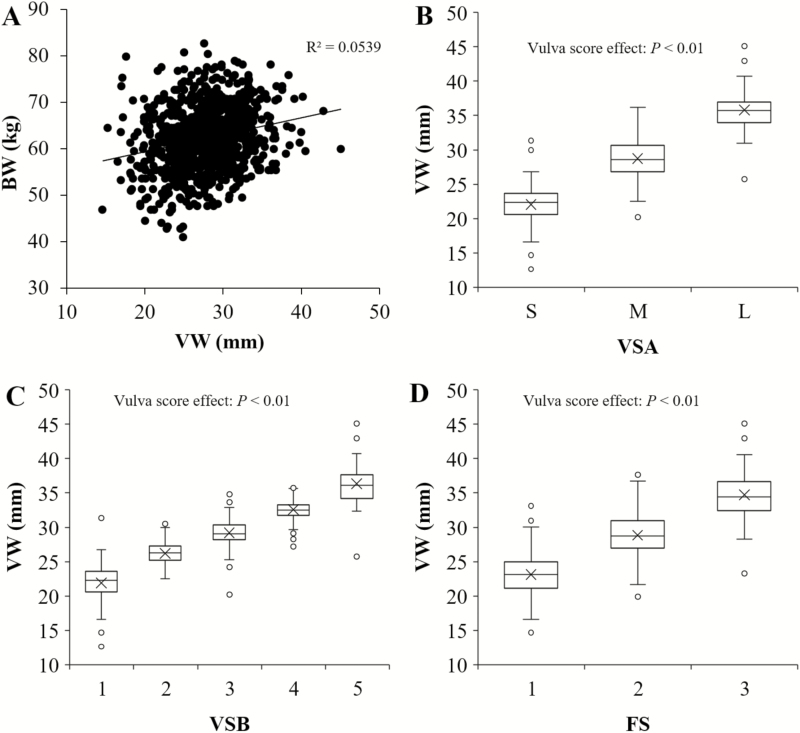
Relationship of vulva width (VW) with body weight (BW) and effectiveness of different vulva scoring methods to accurately partition gilts. (A) BW and VW were assessed at approximately 15 wk of age. A weak positive correlation exists between the 2 traits, with an *R*^2^ value of 0.05 (*P* < 0.01). (B–D) Box and whisker plots of distribution for VW measurements at 15 wk of age based on Vulva Score Method A (VSA; B), Vulva Score Method B (VSB; C), and Farm Score (FS; D). Each method, while variable, was effective in partitioning gilts into separate groups based on vulva size. Points shown beyond the whiskers represent outliers for each score within the scoring method. Whiskers denote the minimum and maximum value for each score while top and bottom boundaries represent the upper and lower quartiles, respectively, with the middle line indicating the median for each score. The X near the median line signifies the mean vulva width measurement for each category. Statistical significance exists for each vulva score mean across methods (*P* < 0.01).

At approximately 15 wk of age, a weak linear association was observed between VW and BW (*R*^2^ = 0.05; *P* < 0.01; Fig. 2A). Average VW measurement for categories within the VSA, VSB, and FS scoring methods differed (*P* < 0.01; Figs. 2B–D, respectively). Across birth week, VW was narrower in birth week 1 relative to birth week 2 or 3 (0.99 and 1.33 mm, respectively; *P* < 0.05; Table 1). Additionally, BW at 15 wk was heavier for birth week 2 compared to birth week 1 or 3 (2.04 and 1.96 kg, respectively; *P* < 0.05; Table 1). Using VSA method, 23.5%, 70.3%, and 6.2% of gilts were distributed into S, M, and L classifications, respectively (Table 2). Similarly, for the VSB method, 22.7%, 25.5%, 34.3%, 12.3%, and 5.3% of gilts were represented in scores 1 to 5, respectively (Table 2). Additionally, the FS method allocated 22.8%, 69.7%, and 7.6% of gilts into vulva score 1 to 3, respectively (Table 2).

**Table 1.** Gilt vulva width (VW) and body weight (BW) means by birth week

	Birth week^1^						
	1		2		3		Total
	*n*	%	*n*	%	*n*	%	*n*
Parameter	194	16.2	490	40.9	274	22.9	958
VW^2^, mm							
Mean	28.72^a^		27.73^b^		27.39^b^		27.58
SEM	0.34		0.18		0.25		0.13
BW^3^, kg							
Mean	61.18^a^		63.22^b^		61.26^a^		61.60
SEM	0.93		0.66		0.97		0.44

^1^Birth week refers to the specific calendar week during which the gilt was born.

^2^VW was measured to the nearest millimeter using digital calipers.

^3^BW was recorded using an individual scale provided at the cooperating gilt development unit (GDU).

^a,b^Differences in letters denote significance level (*P* < 0.05).

**Table 2.** Distribution of gilts by vulva score method

Score	*n*	%
VSA^1^		
S	281	23.5
M	842	70.3
L	74	6.2
VSB^2^		
1	272	22.7
2	305	25.5
3	410	34.3
4	147	12.3
5	63	5.3
FS^3^		
1	162	22.8
2	496	69.7
3	54	7.6

^1^Three scores, small (S), medium (M), and large (L) were assigned using the Vulva Score Method A (VSA).

^2^Five scores, 1–5, were assigned using Vulva Score Method B (VSB).

^3^Three scores, 1–3, were assigned using Farm Score (FS) method.

### Gilts With Increased Vulva Score Have Improved Ability to Achieve Parity 1

Across all vulva scoring methods, gilts scored as S for VSA, 1 for VSB, and 1 for FS, all had decreased likelihood (VSA: 64.7% vs. 73.2%, 84.4%; VSB: 66.0% vs. 71.7%, 79.2%, 76.4%, 84.2%; FS: 67.2% vs. 75.0 %, 88.8%) of reaching P1 when compared to their remaining cohorts within the same scoring method (*P* = 0.02, 0.02, 0.03, respectively). No difference was observed for the percentage of gilts achieving parity 2 (P2) across all scoring methods (*P* = 0.32, 0.29, 0.25). For all scoring methods, no difference was detected in the duration from time of scoring to P1 interval (*P* = 0.43, 0.46, 0.25), first service to P1 interval (*P* = 0.20, 0.66, 0.34), and first service to P2 interval (*P* = 0.32, 0.43, 0.46; Tables 3–5).

**Table 3.** Gilt inclusion rate and comparison of sow productive days using VSA^1^

	Classification				
Breeding parameters	S	M	L	SEM	*P*
Selected gilts^2^	156	530	45		
Gilts achieving parity 1^3^	101	388	38		
% Achieve parity 1^4^	64.7	73.2	84.4		0.02
Gilts achieving parity 2^5^	69	263	25		
% Achieve parity 2^6^	44.2	49.6	55.6		0.32
Score to farrow interval^7^	251.4	252.2	243.3	2.3	0.43
First service to P1 interval^8^	121.5	121.8	116.9	3.1	0.20
First service to P2 interval^9^	269.7	268.8	261.8	3.0	0.32

^1^VSA = Vulva Score Method A.

^2^Gilts that arrived at the designated sow farm gilt development unit (GDU).

^3^Number of gilts successfully producing a first parity.

^4^ *P*-value calculated using chi-square comparison.

^5^Number of gilts who farrowed a second litter.

^6^ *P-*value calculated using chi-square comparison.

^7^Number of days from vulva scoring at the receiving GDU until achieving first parity.

^8^Number of days from first service until first litter farrowing.

^9^Number of days from first service until second litter farrowing.

**Table 4.** Gilt inclusion rate and comparison of sow productive days using VSB^1^

	Classification						
Breeding parameter	1	2	3	4	5	SEM	*P*
Selected gilts ^2^	150	173	264	106	38		
Gilts achieving parity 1^3^	99	124	209	81	32		
% Achieve parity 1^4^	66.0	71.7	79.2	76.4	84.2		0.02
Gilts achieving parity 2^5^	66	77	138	55	21		
% Achieve parity 2^6^	44.0	44.5	52.3	51.9	55.3		0.29
Score to farrow interval^7^	252.0	252.0	252.4	250.0	244.2	3.2	0.46
First service to P1 interval^8^	121.7	119.7	123.2	120.2	117.3	2.4	0.66
First service to P2 interval^9^	269.1	266.0	268.8	272.3	262.4	3.2	0.43

^1^VSA = Vulva Score Method B.

^2^Gilts that arrived at the designated sow farm gilt development unit (GDU).

^3^Number of gilts successfully producing a first parity.

^4^ *P*-value calculated using chi-square comparison.

^5^Number of gilts who farrowed a second litter.

^6^ *P*-value calculated using chi-square comparison.

^7^Number of days from vulva scoring at the receiving GDU until achieving first parity.

^8^Number of days from first service until first litter farrowing.

^9^Number of days from first service until second litter farrowing.

**Table 5.** Gilt inclusion rate and comparison of sow productive days using FS^1^

		Classification			
Breeding parameter	1	2	3	SEM	*P*
Selected gilts^2^	125	384	36		
Gilts achieving parity 1^3^	84	288	32		
% Achieve parity 1^4^	67.2	75.0	88.8		0.03
Gilts achieving parity 2^5^	50	173	21		
% Achieve parity 2^6^	40.0	45.1	58.3		0.25
Score to farrow interval^7^	254.1	252.2	243.3	2.7	0.34
First service to P1 interval^8^	121.6	123.3	117.0	3.5	0.24
First service to P2 interval^9^	266.6	270.4	265.8	3.4	0.46

^1^Farm score (FS) method where trained personnel assigned vulva score.

^2^Gilts that arrived at the designated sow farm gilt development unit (GDU).

^3^Number of gilts successfully producing a first parity.

^4^ *P*-value calculated using chi-square comparison.

^5^Number of gilts who farrowed a second litter.

^6^ *P*-value calculated using chi-square comparison.

^7^Number of days from vulva scoring at the receiving GDU until achieving first parity.

^8^Number of days from first service until first litter farrowing.

^9^Number of days from first service until second litter farrowing.

### P1 and P2 Litter Performance Is Affected by Prepubertal Vulva Score Classification

#### Vulva Score Method A.

First parity TB was 1.2 pigs greater (*P* < 0.01) for gilts scored as M compared to S (Table 6). In contrast to S or M, TB from gilts scored as L were not different, although TB for M and L combined (M+L) was 1.1 pigs greater (*P* < 0.01) relative to gilts with a vulva score S. Differences in P2 performance were detected with fewer (*P* = 0.04) TB between S (12.3) when compared to M (13.6) scored gilts. Furthermore, gilts having a S vulva score produced approximately 1.3 fewer piglets (*P* = 0.02) compared to their remaining cohorts (M+L). Similarly, P2 BA was 1.3 fewer (*P* = 0.02) for gilts whose vulvas scored S compared to their counterparts. Stillborn rate was not affected by vulva score (*P =* 0.24). Expectedly, when total production through P2 was analyzed, TB for M (26.4) and M+L (26.4) combined was greater (*P* < 0.01) compared to gilts having a vulva score S (24.0). The number of BA through P2 for gilts with a vulva score S was 2.0 and 1.9 pigs fewer (*P* = 0.03) compared to gilts with the M and M+L vulva scores, respectively.

**Table 6.** Parity 1 and 2 performance from gilts classified using VSA^1^

	Classification^1^					S vs. M+L	
Parity 1	S	M	L	SEM	P	M+L	P
	(*n* = 100)	(*n* =387)	(*n* = 37)			(*n* = 424)	
Total born	11.79^a^	12.96^b^	12.42^ab^	0.39	0.02	12.91	<0.01
Born alive	10.11	10.85	10.57	0.48	0.35	10.82	0.16
Stillborn	0.43	0.55	0.58	0.10	0.48	0.55	0.24
Mummified	1.09	0.84	0.81	0.19	0.48	0.83	0.20
Parity 2							
	(*n* = 69)	(*n* = 260)	(*n* = 24)			(*n* = 284)	
Total born	12.25^a^	13.62^b^	13.34^ab^	0.50	0.04	13.59	0.02
Born alive	10.86^a^	12.19^b^	11.59^ab^	0.52	0.06	12.14	0.02
Stillborn	0.62	0.73	1.17	0.19	0.24	0.78	0.42
Mummified	0.41	0.37	0.45	0.11	0.88	0.37	0.78
P1 + P2							
	(*n* = 69)	(*n* = 259)	(*n* = 24)			(*n* = 283)	
Total born	23.96^a^	26.44^b^	25.81^ab^	0.74	<0.01	26.38	<0.01
Born alive	21.13^a^	23.11^b^	22.43^ab^	0.81	0.09	23.05	0.03
Stillborn	0.98	1.26	1.65	0.20	0.16	1.30	0.14
Mummified	1.41	1.29	1.16	0.32	0.90	1.27	0.70

^1^Classification assigned using Vulva Score Method A (VSA). Scores Small (S), Medium (M), and Large (L) were assigned based on best fit using VSA scoring tool. M+L represents the combined result of all gilts scored > S.

^a,b^Differences in means denoted with different letters are statistically significant (*P* ≤ 0.05).

#### Vulva Score Method B.

Using VSB to distinguish prepubertal differences in vulva size, P1 TB was greater for gilts receiving either a vulva score of 3 (1.4 pigs) and vulva score of 4 (1.5 pigs) compared to gilts with a vulva score of 1 (*P* < 0.01, Table 7). The P1 TB for all gilts with a vulva score of 2 to 5 were combined (2 to 5) and resulted in increased TB by 1.1 pigs compared to gilts with a vulva score of 1 (*P* < 0.01). Additionally, P1 BA tended (*P* = 0.08) to be greater (1.01 pigs) in gilts with a 3 vulva score compared to gilts with a 1 vulva score. Although other vulva scores were not different, P1 BA was greater (1.1 pig; *P* < 0.01) for gilts with vulva scores 2 to 5 compared to gilts with the assigned vulva score of 1. No vulva score effect was observed for P1 SB (*P* = 0.1) or mummified fetuses (*P* = 0.22). Likewise, SB and MM were not affected when comparing gilts with a vulva score of 1 to the combined vulva scores of 2 to 5. Gilts achieving P2 with a vulva score of 1 produced 1.0 fewer (*P* = 0.02) TB piglets and 1.2 fewer (*P* = 0.03) BA compared to the remaining gilts that scored 2 to 5 (Table 7). Combined TB for P1 and P2 was impacted (*P* < 0.03) by vulva score while total BA through P2 was not (*P* = 0.20). However, total TB and BA for P1 and P2 were increased 2.3 (*P <* 0.01) and 1.9 (*P* = 0.03), respectively, in gilts with a vulva score of 2 to 5 compared to gilts scored 1.

**Table 7.** Parity 1 and 2 performance from gilts classified using VSB^1^

	Classification^1^							1 vs. 2–5	
Parity 1	1	2	3	4	5	SEM	*P*	2–5	*P*
	(*n* = 99)	(*n* = 124)	(*n* = 209)	(*n* = 81)	(*n* = 32)			(*n* = 446)	
Total born	11.75^a^	12.54^ab^	13.14^b^	13.27^b^	11.83^ab^	0.42	0.01	12.85	<0.01
Born alive	10.04	10.47	11.05	11.20	10.33	0.52	0.36	10.83	0.13
Stillborn	0.43^a^	0.43^a^	0.59^abc^	0.34^abd^	0.82^bc^	0.10	0.06	0.51	0.45
Mummified	1.11	0.81	0.79	0.97	0.96	0.22	0.73	0.84	0.22
Parity 2									
	(*n* = 66)	(*n* = 76)	(*n* = 134)	(*n* = 54)	(*n* = 21)			(*n* = 285)	
Total born	12.35	13.40	13.65	13.67	13.83	0.55	0.22	13.30	0.02
Born alive	10.94	12.01	12.16	12.34	12.22	0.58	0.31	12.17	0.03
Stillborn	0.60^a^	0.86^ab^	0.48^a^	0.68^ab^	1.18^b^	0.17	0.07	0.70	0.55
Mummified	0.42	0.49	0.35	0.25	0.51	0.12	0.63	0.37	0.66
P1 + P2									
	(*n* = 65)	(*n* = 76)	(*n* = 136)	(*n* = 54)	(*n* = 21)			(*n* = 287)	
Total born	23.97^a^	25.57^ab^	26.63^b^	26.91^b^	26.36^ab^	0.83	0.03	26.33	<0.01
Born alive	21.11	22.39	23.26	23.75	22.83	0.92	0.20	23.02	0.03
Stillborn	0.96	1.30	1.27	1.11	1.73	0.23	0.35	1.30	0.14
Mummified	1.43	1.10	1.23	1.63	1.30	0.37	0.86	1.27	0.65

^1^Classification assigned using Vulva Score Method B (VSB). Numerical scores (1–5) were assigned based on best fit using VSB scoring tool, with increasing score corresponding to increase in vulva with; 2–5 represents the combined result of all gilts scored > 1.

^a–d^Differences in means denoted with different letters are statistically significant (*P* ≤ 0.05).

#### Farm Score.

Interestingly, no differences were observed across P1 litter data for gilts scored using the FS method (Table 8.). For P2, TB tended to be greater between gilts with vulva score 1 and 3 (1.8 pigs; *P =* 0.06) and between scores 1 and 2 for BA (1.1; *P* = 0.09). When combined totals for scores 2 and 3 were compared to score 1, a tendency for greater TB and BA (1.0, 1.2 pigs; *P <* 0.09, respectively) was observed. When total P1 and P2 production was considered, TB for gilts having a vulva score of 2 or 3 tended to increase compared to gilts with a vulva score of 1 (1.6; *P* = 0.06).

**Table 8.** Parity 1 and 2 performance from gilts classified using FS^1^

	Classification^1^					1 vs. 2–3	
Parity 1	1	2	3	SEM	*P*	2–3	*P*
	(*n* = 84)	(*n* = 288)	(*n* = 32)			(*n* = 320)	
Total born	12.76	12.91	12.83	0.40	0.94	12.90	0.76
Born alive	11.06	10.76	10.93	0.48	0.85	10.79	0.60
Stillborn	0.42	0.55	0.59	0.11	0.47	0.55	0.22
Mummified	0.89	0.87	0.85	0.21	0.99	0.89	0.98
Parity 2							
	(*n* = 50)	(*n* = 173)	(*n* = 21)			(*n* = 194)	
Total born	12.82	13.66	14.60	0.52	0.13	13.78	0.09
Born alive	11.09	12.14	12.64	0.56	0.16	12.24	0.06
Stillborn	0.69	0.70	0.71	0.17	1.00	0.70	0.93
Mummified	0.42	0.36	0.16	0.12	0.49	.32	0.49
P1 + P2							
	(*n* = 50)	(*n* = 173)	(*n* = 21)			(*n* = 194)	
Total born	24.88	26.38	26.81	0.82	0.20	26.51	0.06
Born alive	22.01	22.92	23.75	0.89	.49	23.10	0.25
Stillborn	1.07	1.30	1.51	0.23	0.52	1.32	0.32
Mummified	1.47	1.31	1.25	0.32	0.88	1.29	0.60

^1^Classification assigned using the Farm Score (FS) Vulva Scoring Method. Numerical scores were assigned based on visual appraisal of vulva size with 1 being the smallest, 2 being average, and 3 representing larger vulva size within a contemporary group. 2 + 3 is the combined results of all gilts scoring > 1.

^a–d^Differences in means denoted with different letters are statistically significant (*P* ≤ 0.05).

## DISCUSSION

Maximizing sow retention and reproductive efficiency of selected replacement gilts is essential for productivity, profitability, and farm efficiency. However, multiple genetic elements contributing to reproductive success coupled with a large environmental influence can make selecting replacement gilts with the greatest reproductive potential challenging (Serenius and Stalder, 2006). The age at which a gilt achieves first estrus can be predictive of her longevity and ability to attain later parities (Patterson et al., 2010). Puberty is a critical time-sensitive checkpoint as a gilt’s inability to express standing estrus is a primary cause for failing to enter the breeding herd (Mote et al., 2009). Gilts that reach puberty at an earlier age have an increased ability to return to estrus and ovulate within 10 d after weaning their first parity (Sterning et al., 1998). Indeed, age at first farrowing is a favorable indicator for sow longevity (Hoge and Bates, 2011). Gilts achieving specific reproductive checkpoints (i.e., puberty and parturition) earlier in life are therefore more reproductively capable compared to counterparts achieving the same milestones later in life.

Despite the consensus that early reproductive maturation and puberty onset is associated with improved lifetime productivity in female pigs, a relationship between prepubertal reproductive tract development and subsequent reproductive performance is not well documented in the literature. Previously, our group has demonstrated that VW differences beginning at approximately 95 to 115 d of age was predictive of a gilt’s ability to achieve behavioral estrus by 200 d of age (Graves et al., 2019). Thus, we hypothesized that a positive association between higher VW and parity outcome exists. The study objective was to determine the effectiveness of prepubertal VW scoring as a marker for reproductive tract development, with intent to identify gilts with improved productivity through at least 2 parities.

In Meishan pigs, a breed known for attaining puberty at an early age and producing large litters, follicular development can start as early as 45 d of age (Miyano et al., 1990), indicating hypothalamic-pituitary gonadal axis (HPGx) activity. In the current body of work, gilts were distinguishable by variation in prepubertal VW at approximately 15 wk of age, presumably the result of differential follicular activity and resultant estrogen synthesis and release (Elsaesser et al., 1998). Speculatively, it seems logical that early reproductive tract development would contribute to greater fecundity, potentially due to greater uterine capacity, which is concomitantly associated with decreased embryonic loss (Bolet et al., 1986) and increased pigs produced though 4 parities (Freking et al., 2016). While we did not measure uterine capacity per se, this study revealed that gilts with larger vulvas at 15 wk of age had greater TB and BA through 2 parities.

Based on the results herein and our previous findings (Graves et al., 2019), vulva score variation at 15 wk of age could result from differential endogenous estrogen production from tertiary follicle development. Estrogen activity, mediated through its multiple receptors, is essential for regulating reproductive function in the pig, providing HPGx regulation as well as the signal for pregnancy recognition (Dhindsa and Dziuk, 1968; Perry et al., 1973; Bazer and Thatcher, 1977; Geisert et al., 1982). Additionally, the estrogen receptor locus is a major contributor to litter size in pigs (Rothschild et al., 1996). Additionally, the pig uterus increases in size and growth rate at approximately 80 d of age in response to ovarian-produced estrogens (Dyck and Swierstra, 1983). Our results suggest that VW is also responsive to ovarian estrogen production in pigs. This is plausible as the gilt vulva is responsive to estrogenic compounds, including mycotoxins has been documented (Dacasto et al., 1995, Jiang et al., 2011). This response potentially indicates earlier HPGx activation. Based on the previous work demonstrating the importance of estrogen and its receptors (Bazer and Thatcher, 1977; Rothschild et al., 1996), particularly with respect to establishing the cross communication between the dam and conceptus (Geisert et al., 1982; Morgan et al., 1987), it seems plausible that gilts with increased production of and sensitivity to estrogen may have a reproductive advantage. This speculation warrants further investigation to understand the relationship between reproductive tract responses to estrogen and its effects on fertility.

## CONCLUSION

Selection for reproductively superior replacement gilts is challenging. Gilts with reduced vulva score at 15 wk of age had lower inclusion rate into the breeding herd and produced fewer pigs through 2 parities. Activation of the HPGx precedes the onset of puberty and these data suggest that relative differences in vulva score, as a proxy for ovarian estrogen synthesis, prior to puberty onset at 15 wk of age is predictive of future reproductive performance. Thus, vulva score classification at an appropriate age could be a valuable tool for identifying gilts with the greatest reproductive potential.
